# Central nervous system Tuberculosis in a man from Cambodia with worsening headaches

**DOI:** 10.1002/ccr3.1119

**Published:** 2017-08-15

**Authors:** Daniel S. Krauth, Kristi K. Stone‐Garza, Deirdre E. Amaro, Sharon L. Reed, Theodoros F. Katsivas

**Affiliations:** ^1^ Department of Medicine Naval Medical Center San Diego San Diego CA USA; ^2^ Department of Medicine Division of Infectious Diseases Naval Medical Center San Diego San Diego California; ^3^ Department of Pathology Neuropathology University of California San Diego California; ^4^ Department of Medicine Pathology University of California San Diego La Jolla California; ^5^ Department of Medicine Divisions of General Internal Medicine and Infectious Disease University of California La Jolla San Diego California

**Keywords:** CNS TB, disseminated TB, tuberculoma

## Abstract

Central nervous system (CNS) tuberculosis should be considered in patients from endemic nations with worsening neurological symptoms. If imaging reveals possible CNS tuberculomas, potentially life‐threatening lesions should be excised and analyzed. When disease is less severe, other tissues possibly infected should be biopsied first for diagnosis to avoid neurosurgery.

## Introduction

Central nervous system (CNS) tuberculosis (TB) is a potentially devastating disease and may require rapid medical or surgical intervention. CNS TB with focal neurological deficits is suggestive of a tuberculoma or abscess; however, nonfocal neurological deficits, such as encephalopathy, are more suggestive of TB meningitis. The medical treatment for CNS TB has remained largely unchanged for decades 1, and the antituberculosis therapy (ATT) regimens for tuberculomas and TB meningitis are the same 2. Neurological surgery, however, is reserved for special cases in the treatment and diagnosis of the former.

Indications for surgery are often due to life‐threatening mass effect from tuberculomas or vasogenic edema, or when the precise etiology of the neurological disease is first unclear.

Here, we discuss a case of successful neurosurgical and medical management of a patient with CNS TB where urgent intervention was indicated, as well as circumstances where tuberculomas may be managed without neurosurgery.

## Case History

**Figure 1:**
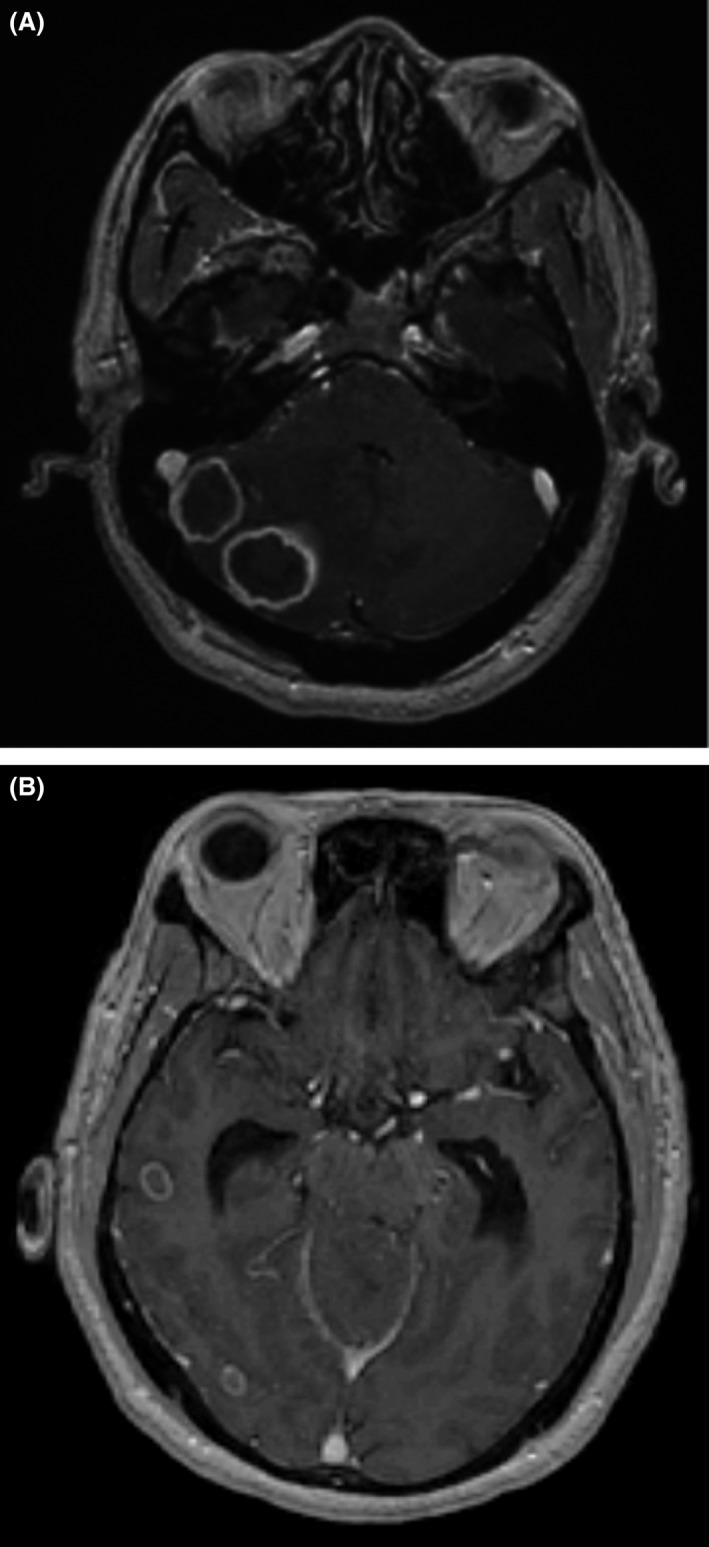
3D Fast Spoiled Gradient‐Recalled‐Echo MRI demonstrating infratentorial ring‐enhancing lesions in the right cerebellum with surrounding vasogenic edema and consequential partial effacement of the fourth ventricle (A), as well as additional lesions in the right temporal lobe (B).

**Figure 2:**
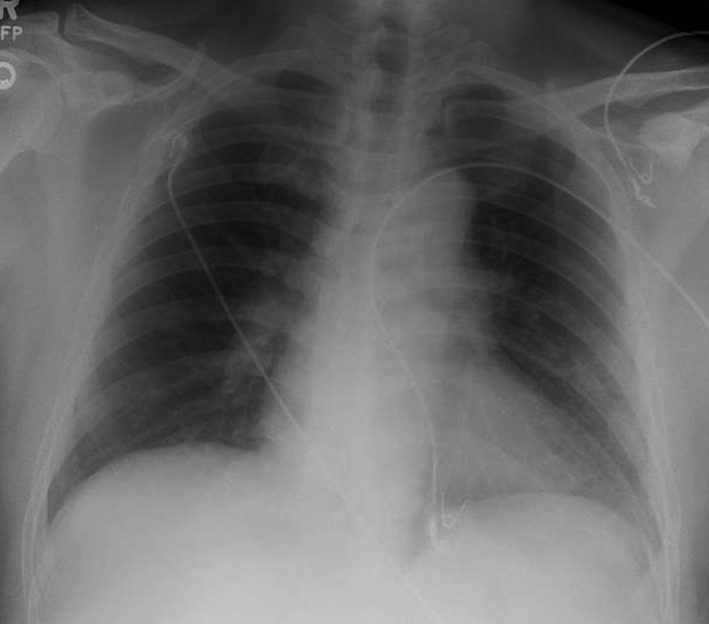
Anterior–posterior chest X‐ray demonstrating diffuse opacifications in the left middle and lower lung fields.

A 61‐year‐old man from Cambodia was referred to the neurosurgery outpatient clinic for 4 years of worsening headaches, imbalance, vomiting, and abnormal findings on a head CT. In Cambodia, he worked on farms with livestock and in rice fields for several years and eventually retired as an office administrator. He moved to the United States 8 years ago, living 1 year in Chicago and for 7 years in San Diego as a “box” factory worker. He has a remote 30 pack‐year history of smoking. He was afebrile and appeared well. His head and neck examination demonstrated pupils equally round and reactive to light. Breath sounds were normal and without crackles or crepitations. On neurological examination, his cranial nerves were intact, he had no nystagmus, and his tandem gait was normal. Dysdiadochokinesia was not observed; however, right‐sided dysmetria was revealed. His complete blood count and electrolytes were normal, his glycated hemoglobin was <6%, and his fasting blood sugar was <100 mg/dL; cysticercosis, toxoplasmosis, and HIV serologies were negative; and interferon‐gamma release assay was indeterminate. He received a MRI with contrast (Fig. 1) of his head that revealed multiple ring‐enhancing lesions and surrounding vasogenic edema in right temporal lobe parenchyma with prominent lesions in the right cerebellar hemisphere, crowding of the basal cisterns and prominent lateral ventricles and was referred to the neurosurgery clinic. Chest X‐ray demonstrated opacifications in the left mid‐ and lower lung (Fig. 2).

**Figure 3:**
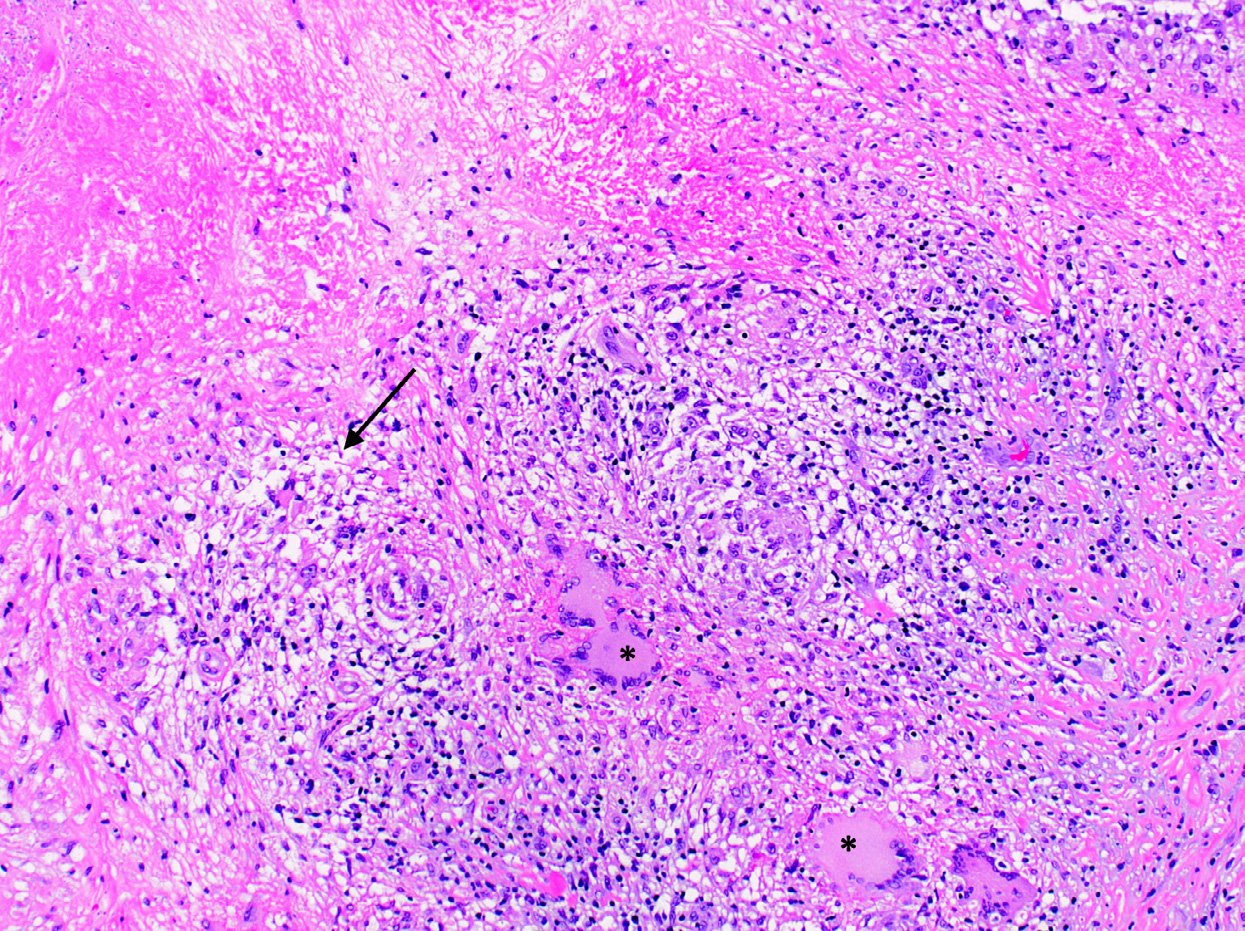
Chronic granulomatous inflammation demonstrated by necrosis (arrow), multinucleated giant cells (asterisks), and chronic lymphogenic infiltration. H&E stain at 100×.

**Figure 4:**
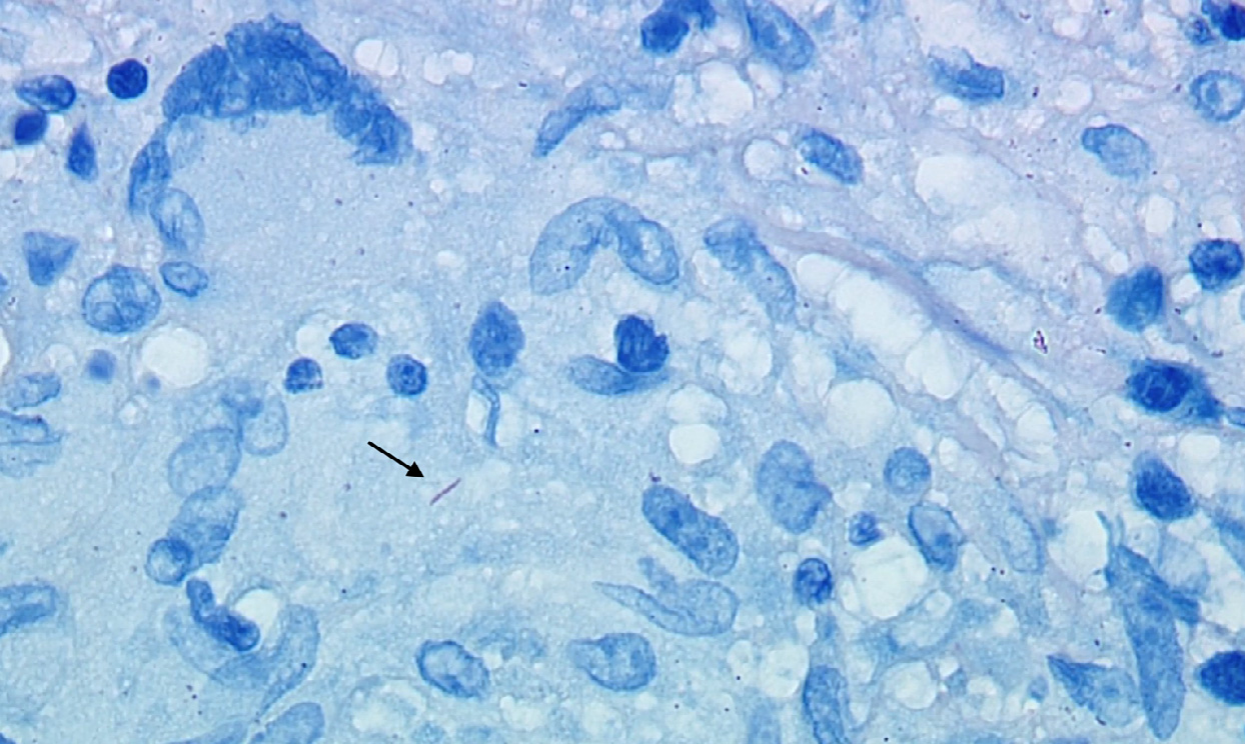
MTB within a multinucleated giant cell (arrow). AFB stain at 100×.

The patient underwent excision of the cerebellar masses, which appeared grossly purulent. Brain pathology revealed necrotizing granulomatous inflammation (Fig. 3) and rare acid‐fast‐positive bacilli (Fig. 4). Brain tissue was assayed for *Mycobacterium tuberculosis* (MTB) by nucleic acid amplification (NAA) testing with GeneXpert and was positive without evidence of rifampin resistance. CT scan of the chest, abdomen, and pelvis revealed diffuse nodularity in the lungs and abdominal omentum. Cultures of brain tissue eventually grew MTB susceptible to isoniazid, rifampin, ethambutol, and pyrazinamide. CSF, stool, urine, and sputum cultures, however, were negative for MTB after 8‐week incubation.

## Discussion

In the immune competent, the differential diagnosis for cerebral ring‐enhancing lesions includes tuberculomas, neurocysticercosis, melioidosis, glioblastomas, metastases, subacute hemorrhagic strokes, and demyelinating diseases such as acute disseminated encephalomyelitis.

CNS TB requires a high level of suspicion and should be considered in patients with origins from a TB endemic nation even in the absence of pulmonary symptoms. As a form of disseminated TB, tuberculomas are a result of hematogenous or lymphogenic spread 2, 3.

During work‐up, CSF studies are often not acquired due to the risk of herniation with lumbar puncture. If CSF circulation appears to be obstructed due to mass effect on imaging, as in the case described here, or if focal deficits are present on examination, craniotomy with neurosurgical debulking is appropriate prior to preemptive ATT. If non‐CNS tissue with possible disease can be safely attained by biopsy, it should be acquired and tested for the presence of TB (AFB stain or culture, or NAA testing), and if TB is identified, intracranial lesions may be preemptively treated with ATT 3, 4. However, if only CNS lesions are detected, stereotactic biopsy is generally indicated to make the diagnosis 3, 5.

A standard treatment course for tuberculomatous disease with or without meningitis is 12 months 6. Due to poor CNS penetration with ethambutol, a fluoroquinolone (moxifloxacin or levofloxacin) or an injectable aminoglycoside is often added during the intensive 4‐drug phase while TB drug susceptibilities are pending. If the TB is sensitive to standard ATT, then the treatment regimen may be narrowed to three medications: rifampin, isoniazid, and pyrazinamide for the remainder of the 2‐month “intensive phase” 6, 7. With susceptible MTB isolates, isoniazid and rifampin daily or three times weekly for at least 10 months thereafter are adequate for completion of standard 12‐month therapy. Additionally, a glucocorticoid burst and taper at the initiation of ATT reduces the risk of paradoxical worsening of symptoms from inflammation and has demonstrated improved long‐term function with increased rates of survival during the treatment of CNS TB 8, 9. Concerning our patient, he tolerated and completed the above‐described treatment regimen well without complications after surgery. His headache and nausea have resolved; however, his right‐sided dysmetria persists.

Some suggest treating until resolution of radiographic evidence of lesion enhancement or edema from tuberculomas is achieved 10, 11. However, CDC guidelines do state that 12 months of ATT for tuberculomas is sufficient 6. Regardless of the decided duration of therapy, the initial clinical approach to a clinically stable patient with lesions suggestive of CNS tuberculomas and with known risk factors for TB involves a survey for extracranial lesions that may be biopsied and analyzed for TB in order to guide empiric treatment of the intracranial disease with close clinical observation. However, if the intracranial lesions are potentially life‐threatening, neurosurgical intervention may be appropriate for initial treatment as well as a more rapid diagnosis.

## Authorship

DSK: contributed to the evaluation and management of the patient and wrote the manuscript in its entirety; KKS: contributed to the evaluation and management of the patient; DEA: contributed the neuropathology images and image descriptions; SLR: contributed the microbiology assays and AFB stains; TFK: supervised the evaluation of the management of the patient as well as expert critiquing of the manuscript.
